# Tanshinone IIA changed the amniotic fluid volume and regulated expression of AQP1 and AQP3 in amniotic epithelium cells: a promising drug treating abnormal amniotic fluid volume

**DOI:** 10.1186/s10020-023-00687-6

**Published:** 2023-06-29

**Authors:** Shuangjia Pan, Yehui Lan, Baoyi Chen, Yujia Zhou, Xinxin Ying, Ying Hua

**Affiliations:** Department of Obstetrics and Gynecology, The Second Affiliated Hospital of Wenzhou Medical University, Wenzhou, 325027 China

**Keywords:** Tanshinone IIA, Abnormal amniotic fluid volume, Aquaporins, Fetal membranes, Glycogen synthetic kinase 3β, AQP1 knockout

## Abstract

**Background:**

Many studies have confirmed the association of aquaporins (AQPs) with abnormal amniotic fluid volume (AFV). In our previous experiments, we found that Tanshinone IIA was able to regulate the expression of AQP1 and AQP3. However, the exact mechanism by which Tanshinone IIA regulates AQPs protein expression and its effect on AFV remains unclear. The purpose of this study was to investigate the effects of Tanshinone IIA on AFV and the possible molecular mechanism of regulation of AQP1 and AQP3.

**Methods:**

The expression of AQPs protein in the amniotic membranes was compared between pregnant women with normal pregnancy and those with isolated oligohydramnios. The AQP1 knockout (AQP1-KO) mice and wild-type (WT) mice were treated with saline or Tanshinone IIA (10 mg/kg) at 13.5GD and 16.5GD. Human amniotic epithelium cells (hAECs) from pregnant women with normal AFV and isolated oligohydramnios were incubated with 35 μmmol/L Tanshinone IIA or 25 mmol/L LiCl [inhibitor of glycogen synthetic kinase 3β (GSK-3β)]. The protein expressions of AQPs, GSK-3β, phospho-GSK-3β (Ser9) in fetal membranes of mice and human amniotic epithelium cells were detected by western blotting.

**Results:**

The expression of AQP1 protein in the amniotic membrane of isolated oligohydramnios was increased compared with normal pregnancy. The AFV in AQP1-KO mice is higher than that in WT mice. In wild-type mice, AFV in Tanshinone IIA group was significantly higher than that in control group, and AQP1 protein expression was significantly lower than that in control group, but in AQP1 knockout mice, Tanshinone IIA reduced amniotic fluid volume and AQP3 protein expression at 16.5GD. Tanshinone IIA reduced AQP1, AQP3 and p-GSK-3β (Ser9) protein expression in normal hAECs, and this effect was inhibited by LiCl. In hAECs with oligohydramnios, the down-regulation of AQP1 and up-regulation of AQP3 by Tanshinone IIA was independent of GSK-3β signaling pathway.

**Conclusions:**

Tanshinone IIA may increase AFV in normal pregnancy by downregulating AQP1 protein expression in the fetal membranes, which may be associated with p-GSK-3β signaling pathway. But a larger AFV in AQP1-KO mice was significantly attenuated by Tanshinone IIA, which may be related to AQP3. Tanshinone IIA is a promising drug for the treatment of amniotic fluid abnormality.

## Background

The amniotic fluid provides the environment necessary for fetal development (Beall et al. 2007a).The intramembrane absorption of the amniotic fluid maintains the stability of amniotic fluid (Beall et al. 2007a). Oligohydramnios is defined as amniotic fluid volume (AFV) of less than 300 ml during the third trimester, while polyhydramnios is defined as AFV of more than 2000 ml (Croom et al. 1992). Oligohydramnios or polyhydramnios can lead to adverse outcomes in newborns (Ozgen et al. 2021). However, the etiology of many clinical cases of amniotic fluid abnormalities is unknown, and it is absent of effective treatment.

Aquaporins (AQPs), a membrane protein with a molecular weight of 25–34 kDa, can increase the water permeability of the lipid bilayer of cell membranes (Carbrey and Agre 2009). To date, 13 AQP isoform (AQP 0–12) have been identified in mammals (Shao et al. 2021). AQP1, AQP3, AQP8, AQP9 have been found to be expressed in amniotic membrane of human, mice and other mammalians, playing an important role in the intramembrane transport of amniotic fluid (Mann et al. 2005). The expression levels of AQPs varied with amniotic fluid volume disorders (Mann et al. 2002; Damiano et al. 2001; Aralla et al. 2012; Bednar et al. 2015; Zhu et al. 2015). 13 AQPs can be divided into three subfamilies according to their function. The first group is called aquaporins, rapidly transporting water across the cell membrane only, including AQP0, AQP1, AQP2, AQP4, AQP5, AQP6 and AQP8. The second group is called aquaglyceroporins, active in the transport of both water and small nonpolar molecules like glycerol and urea, including AQP3, AQP7, AQP9 and AQP10. The last one is called superaquaporin, including AQP11 and AQP12, they are found to transport water, glycerol, and H2O2 (Pérez-Pérez et al. 2020). Some studies have shown that compared to wild-type (WT) mice, AQP1 knockout mice showed a significant increase in amniotic fluid volume and a decrease in amniotic fluid osmolality (Mann et al. 2006; Luo et al. 2018), while AQP3 knockout mice were associated with reduced amniotic fluid volume and fetal growth (Seo et al. 2018). Hence, the change of AQP1 and AQP3 expression was closely associated with the abnormal amniotic fluid volume.

Tanshinone IIA is an effective active ingredient extracted from Salvia miltiorrhiza. Due to its anti-inflammatory, antioxidant and anti-tumor effects, Tanshinone IIA has been widely used in the treatments of various diseases, such as heart disease, cerebrovascular disease, hepatitis and acute lung injury (Zhou et al. 2005; Ansari et al. 2021; Quan et al. 2019). Li et al. found that Tanshinone IIA could relieve seawater immersion-induced lung injury by inhibiting the expression of AQP1 and AQP5 proteins in alveolar cells (Li et al. 2011). It has also been reported that Tanshinone IIA alleviated cerebral edema caused by brain injury through the downregulation of AQP4 (Huang et al. 2020). Thus, the therapeutic effect of Tanshinone IIA may be related to its regulation of AQPs.

Glycogen synthase kinase-3β (GSK-3β) signaling pathway plays a role in many cellular processes and is therapeutic targets for type 2 diabetes, neurodegenerative diseases, and inflammation (Choi et al. 2020; Lin et al. 2020). The activity of GSK-3β is determined by site-specific phosphorylation and is inhibited by phosphorylation of Ser9 on GSK-3β (Yan et al. 2019). Our previous study showed that Tanshinone IIA regulated AQPs expression in human amniotic epithelial WISH cells, which was associated with GSK-3β signaling pathway (Hua et al. 2017).

However, whether Tanshinone IIA changes amniotic fluid volume, and whether the change is related to the expression of AQP1 and AQP3 in the fetal membrane, and whether the GSK-3β signaling pathway is involved in these relationships. All these questions remain unclear. Therefore, this study first identified the differences in AQP1 and AQP3 protein expression between the women with normal pregnancy and those with isolated oligohydramnios. Second, the effects of AQP1 knockdown on AFV and the expression of AQP3 protein were observed. Third, we analyzed that the effects of Tanshinone IIA on AFV and AQP1 and AQP3 protein expression in fetal membrans of AQP1-KO pregnancy mice. Finally, the relationship between Tanshinone IIA regulating AQP1 and AQP3 protein expression and GSK-3β signaling pathway was explored in human amniotic epithelium cells (hAECs). The purpose of our study was to investigate the effects of Tanshinone IIA on AFV and AQPs protein expression and the possible molecular mechanisms through in vitro and in vivo experiments, which may provide a novel strategy for the treatment of abnormal AFV in pregnant women.

## Methods

### Reagents

Tanshinone IIA was from Shanghai Yuanye Biotechnology Co., Ltd. and LiCl was from Beijing Solaibao Biotechnology Co. Primary antibodies against AQP1 and AQP3 were from Abcam, UK; DNA extraction kit, AQP1 gene upstream universal primer 1, knockout region primer 2, and replacement fragment primer 3 were from Shanghai Biotech Bioengineering Co.

### Animal experiments

Healthy 6–8 weeks-old AQP1 heterozygous mice were from Shanghai Southern Model Biotechnology Co. After approval of the experimental protocol by the Experimental Ethics Committee of Wenzhou Medical University (No. wydw2019-0260), all mice were housed in SPF-grade animal rooms with standard lighting (12 h light, 12 h dark), temperature (23 ± 1 °C), and humidity (50–60%), and had free access to a nutritionally balanced diet and drinking water. AQP1 heterozygous mice were mated to obtain offspring whose genotypes were determined by genetic identification. The male and female mice were caged together in a ratio of 1:2. Timing of pregnancy was determined by visual inspection of the vaginal plug, which was defined as day 0.5 of pregnancy.

AQP1 knockout and wild-type pregnant mice were intraperitoneally treated with saline (n = 6) or Tanshinone IIA (10 mg/kg) (n = 6) from 9.5 GD to 13.5 GD or 16.5 GD once a day. Tanshinone IIA (10 mg/kg) dissolved in 0.1% solution of DMSO. Saline containing an equal amount of DMSO was used as a vehicle control. Fetal membrane and amniotic fluid were collected. AFV, fetal weight and placental weight, as well as the number of fetal mice and embryonic atrophy were recorded.

### Case–control study

All human amnions (n = 34) were from the Department of Obstetrics and Gynecology of the Second Affiliated Hospital of Wenzhou Medical University. The amnions were collected from singleton term pregnancy after cesarean delivery. The amnions with pregnancy complications such as diabetes mellitus, hypertension, cardiovascular disease and autoimmune disease, as well as those with a history of drug induced labor, drug use, premature rupture of membranes, overdue pregnancy, fetal growth restriction, and fetal malformations were excluded in this study.

The first step was to determine abnormal amniotic fluid volume by ultrasound test before delivery. Pregnant women with amniotic fluid index (AFI) of 8–25 cm were included in the normal amniotic fluid group. Pregnant women with the single deepest fluid pocket (SDP) ≤ 2 cm or AFI ≤ 5 cm were included in the oligohydramnios group. The second step was to verity the amount of amniotic fluid during the cesarean section. Mothers with amniotic fluid volume of 300–2000 ml were finally enrolled in the normal amniotic fluid group (n = 20), and those with amniotic fluid volume < 300 ml were enrolled in oligohydramnios group (n = 14). This study was approved by the Research Ethics Committee of the Second Affiliated Hospital of Wenzhou Medical University (No.2016-28) and all mothers signed written informed consents before delivery.

### Cell culture and treatment

The human amniotic membrane is removed aseptically after delivery and stored in Dulbecco’s Modified Eagle Medium (DMEM) containing 10% Penicillin–Streptomycin solution. Human amniotic epithelial cells (hAECs) were obtained by digestion of amniotic membrane with 0.25% trypsin − 0.02% EDTA digestive solution. Then, hAECs were cultured in DMEM containing 10% fetal bovine serum and 1% Penicillin–Streptomycin solution, and were placed in a humidified incubator with 5% CO_2_ and a temperature of 37 °C. When reaching 80–90% confluency, cultured cells were passaged. To explore the effect of Tanshinone IIA on AQPs and its relationship with GSK-3β, hAECs were incubated with complete medium containing 0.1% DMSO, 35 μM Tanshinone IIA, 25 mM LiCl and 35 μM Tanshinone IIA + 25 mM LiCl for 24 h. Their concentrations in medium were based on our previous study (Hua et al. 2017). Tanshinone IIA was dissolved in 0.1% solution of DMSO. All experiments were performed in triplicate.

### Identification of mice genotypes

Using 5′-ACTCAGTGGCTAACAACAAACAGG-3′, 5′-AAGTCAACCCTCTCT GCTCAGCTGGG-3′, 5′-CTCTATGGCTTCTGAGGCGGAAAG-3′ primers, the genotypes of the mice were verified by polymerase chain reaction according to the protocol of the reagent vendor. The 400 bp and 500 bp PCR products were generated from the zero allele and wild-type allele, respectively.

### Western blotting

Total proteins from amniotic membrane and cells are extracted using RIPA lysis buffer containing a mixture of protease inhibitors. Protein concentrations were measured using the BCA Protein Assay Kit according to the manufacturer’s protocol. After boiling the total protein, the proteins (40 μg per well) were separated in 10% SDS-PAGE and then transferred to PVDF membranes. To seal the proteins, the PVDF membranes were incubated with 5% fat-free milk for 90 min. Then, the membranes were incubated overnight at 4 °C using primary antibodies AQP1 (1:1000, Abcam, UK), AQP3 (1:1000, Abcam, UK), GSK-3β (1:1000, Affinity, China), p-GSK-3β (Ser9) (1:1000, Affinity, China), Tubulin (1:3000, Affinity, China). After washing, the membrane was incubated with the appropriate secondary antibody (horseradish peroxidase–conjugated goat anti-rabbit IgG (1:3000, Biosharp, China) and anti-mouse IgG (1:3000, Biosharp, China)) for 2 h. After detection of the signal using digital imaging equipment, the protein bands were quantified by Image-Pro Plus software.

### Immunohistochemistry

The amniotic membrane tissues were fixed in 4% paraformaldehyde, paraffin-embedded, and then sliced into 5-μm sections sections. After deparaffinization, rehydration and antigen retrieval, nonspecific binding of sections were blocked with goat serum. Subsequently, the sections were incubated with rabbit polyclonal antibody AQP1 (1:100, Affinity, China), AQP3 (1:200, Boster, China) primary antibody overnight at 4 °C. The tissues were then incubated with secondary antibody at 37 °C for 40 min, followed by incubation with diaminobenzidinex as a chromogen. Images were assessed using an Olympus optical microscope (Japan). Scoring standard: according to the staining intensity of positive cells, 0 for non-staining, 1 for light yellow, 2 for brown, and 3 for brown yellow. According to the positive detection rate of positive cells, 0 for 0–5%, 1 for 6–25%, 2 for 26–50%, 3 for 51–75%, and 4 for higher than 75%. The immunohistochemistry (IHC) scores were evaluated by multiplying the 2 scores. Five 200-fold visual fields for each sample were randomly selected, and the average score of the five visual fields is the final score of the sample.

### Immunocytochemistry

HAECs were seeded on cover glasses placed in 6-well plates. When reaching 70% confluency, the cellular slides were fixed with 4% paraformaldehyde. After increased the permeability and inactivated endogenous peroxidase, the cellular slides were incubated with Cytokeratin18 (1:200, ABclonal, China) primary antibody overnight at 4 °C. Then, the cellular slides were incubated with biotinlabeled goat anti-rabbit IgG antibody (1:200, ZSGB-BIO, China) for 30 min at 37 °C. DAB kit (ZSGB-BIO, China) was used to visualize staining. Negative controls were performed by using normal rabbit serum instead of the primary antibody. Images were collected using an Olympus optical microscope (Japan).

### Statistical analysis

All data were expressed as mean ± standard deviation or median (interquartile range) and were repeatedly analyzed using SPSS 26.0 statistical software. Most of the information conformed to normal distribution between the two groups, and the t-test was used for data comparison. The rank sum test was used for data comparison between the two groups whose parity and gravidity did not conform to normal distribution information. One-way ANOVA with LSD post hoc was used for comparison among groups, and Dunnett’s T3 method was used for multiple group comparisons with uneven variance. The differences in the rate of copulation plug, successful pregnancies and atrophied embryos in mice between the two groups were analyzed using a four-cell table chi-square test. *P* < 0.05 was considered a statistically significant difference.

## Results

### AQP1 protein expression in the amniotic membrane was increased in isolated oligohydramnios group vs normal AFV group

**Fig. 1:**
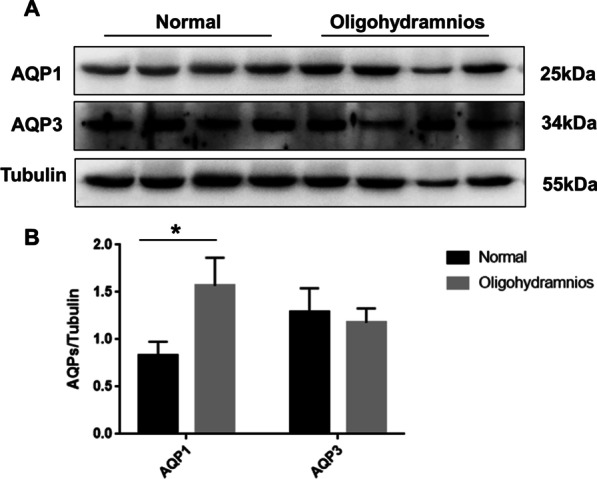
AQP1 expression in oligohydramnios group was significantly higher than that in normal AFV group. **A** Western blotting of AQP1, AQP3 protein expression in amniotic membrane. **B** Quantification of the expressions of AQP1, AQP3 relative to Tubulin. * represents significant difference compared with normal AFV and oligohydramnios group, *P* < 0.05

**Fig. 2:**
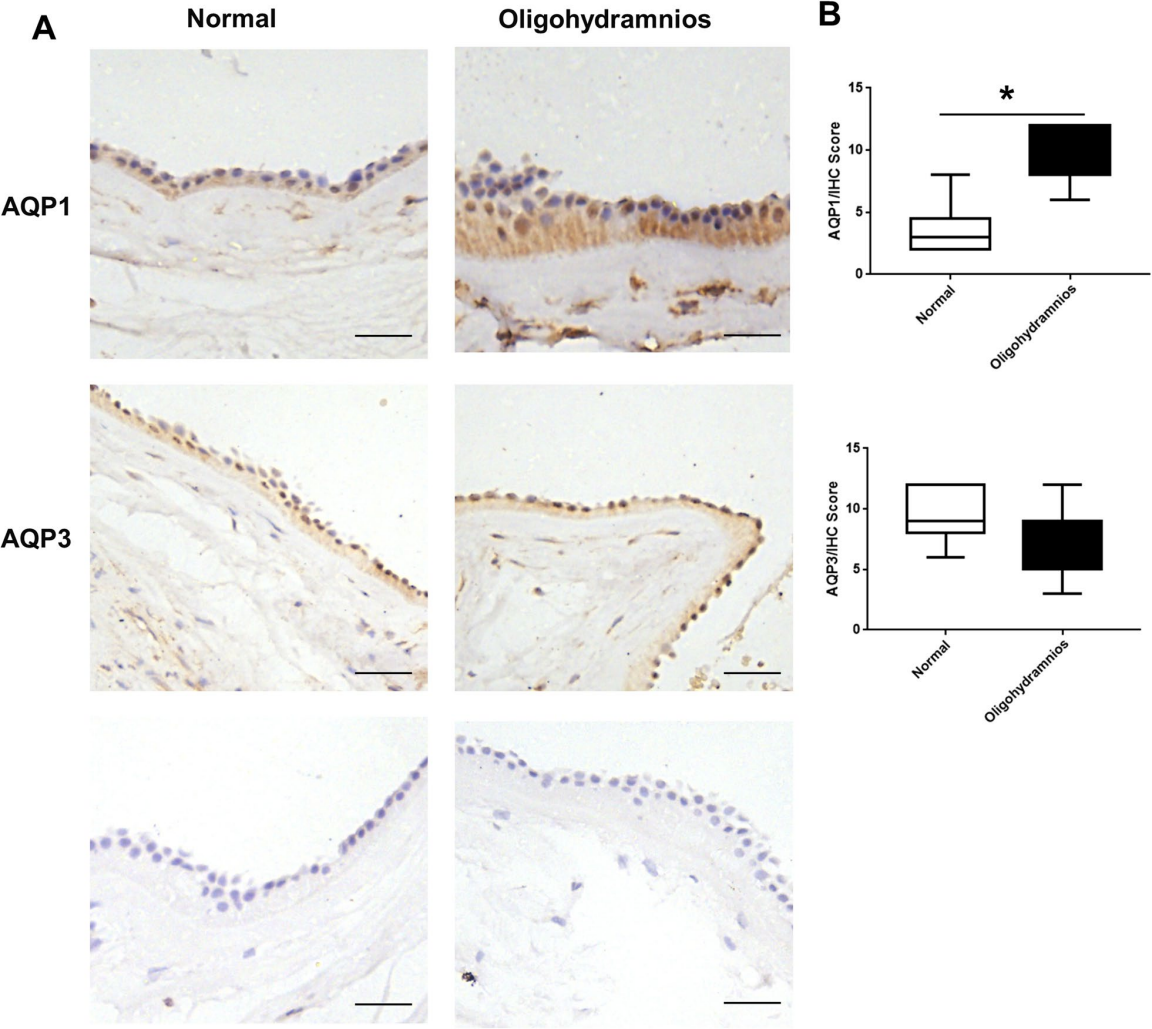
**A** Immunohistochemistry of AQP1, AQP3 protein expression in amniotic membrane. **B** Quantification of IHC score of AQP1, AQP3 expressions in amniotic membrane. AQP1 and AQP3 were localized in the plasma membrane and cytoplasm of human amniotic epithelial cells. Data were analyzed by ANOVA. * represents significant difference compared with normal AFV and oligohydramnios group, *P* < 0.05. IHC, immunohistochemistry. Bar, 20 μm

To investigate the differences of AQP1 and AQP3 expressions in human amniotic membrane between the normal AFV group and the oligohydramnios group, 20 cases were included in the normal AFV group and 14 cases were included in the isolated oligohydramnios group according to the criteria. There were no statistical differences in maternal age, gestational week, fetal weight, gravidity and parity between the two groups (Table 1). Compared with the normal AFV group, the expression of AQP1 protein in amniotic membrane were increased in the oligohydramnios group (Figs. 1, 2), but no significant difference was found in AQP3 expression between the groups (Figs. 1, 2). AQP1 and AQP3 were localized in the plasma membrane and cytoplasm of human amniotic epithelial cells (Fig. 2). Therefore, AQP1 protein expression in the amniotic membrane was increased in pregnant women with isolated oligohydramnios.

**Table 1:** Comparison of the baseline characteristics between the normal AFV group and isolated oligohydramnios group

Group	Number of cases	Age (year)	Gestational Week (week)	Fetal weight (g)	Gravidity (times)	Parity (times)
Normal AFV group	20	31.40 ± 4.64	37.85 ± 1.27	3243.50 ± 418.32	3 (2,4)	1 (0,1)
Isolated oligohydramnios group	14	31.14 ± 4.76	38.21 ± 1.19	3142.14 ± 372.29	3 (1,3)	1 (0,1)
*P*		0.876	0.404	0.473	0.641	0.436

*AFV* amniotic fluid volume

Data were compared by Chi-square test for categorical variables or student t-test for quantitative variables, values are reported as mean ± SD or n (%)

### AFV increased in AQP1 knockout mice

**Fig. 3:**
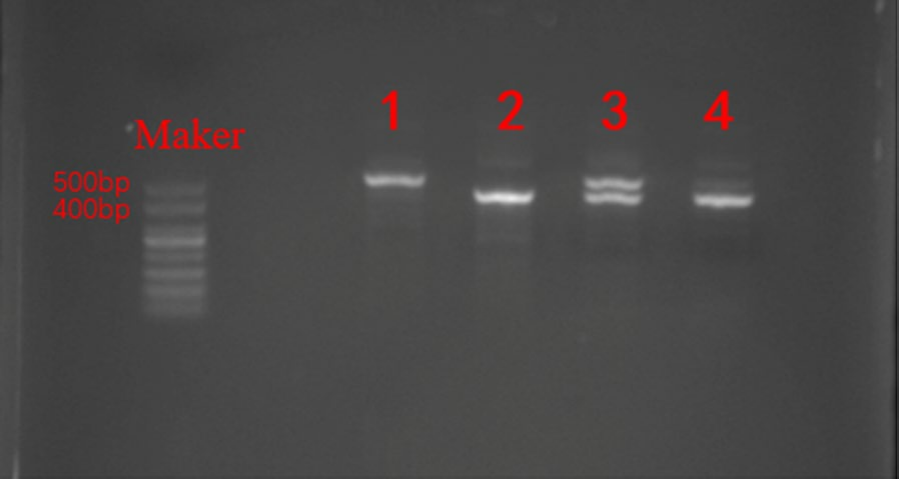
The genotype of mice. In agarose gel electrophoresis, the presence of 400 bp, 500 bp, both 400 and 500 bp bands indicated that the genotypes of mice were AQP1-KO, WT and AQP1 heterozygous, respectively

To investigate the relationship between the decreased AQP1 expression and amniotic fluid volume, AQP1 knockout mice were compared with wild-type mice. Identification of mice genotypes by agarose gel electrophoresis (AGE) showed that the genotype of mice number 1 is WT, the genotype of mice number 2 and number 4 is AQP1-KO, and the genotype of mice number 3 is AQP1 heterozygous (Fig. 3).

**Fig. 4:**
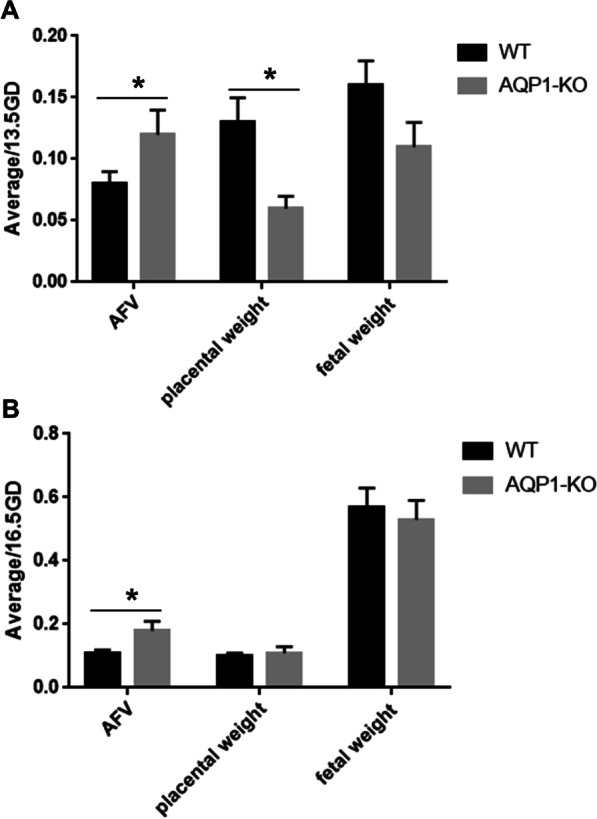
AFV was significantly increased in AQP1-KO mice compared with WT mice. **A** AFV, fetal weight, and placental weight in the WT group and AQP1-KO group at 13.5GD. **B** AFV, fetal weight, and placental weight in the WT group and AQP1-KO group at 16.5GD. * represents significant difference compared with WT group and AQP1-KO group, *P* < 0.05. *AFV* amniotic fluid volume

Compared with WT pregnant mice, AQP1-KO pregnant mice have a significantly lower copulation plug rate, while AQP1 knockdown had no effect on successful pregnancy rates of mice (Table 2). Compared with WT mice, AFV of AQP1-KO mice was dramatically increased whether at 13.5GD or at 16.5GD (Fig. 4). AQP1 knockdown had no significant effect on fetal weight and placental weight compared to WT mice at 16.5GD (Fig. 4).

**Table 2:** Comparison of atrophied embryos rate, copulation plug rate and successful pregnancy rate between AQP1(−/−) and AQP1(+/+) mice

	AQP1(−/−)		AQP1(+/+)		*P*
	Control group	Tanshinone IIA group	Control group	Tanshinone IIA group	
Atrophied embryos rates n (%)	N^a^ = 50	N^a^ = 54	N^a^ = 74	N^a^ = 97	0.094
	8 (16.0)	10 (18.5)	4 (5.4)	10 (10,3)	
Copulation plug rates n (%)	N^b^ = 57		N^b^ = 59		0.000*
	33 (67.9)		55 (93.2)		
Successful pregnancy rates n (%)	N^b^ = 33		N^b^ = 55		0.431
	24 (72.7)		44 (80.0)		

^a^Refers to the total number of embryos

^b^Refers to the total number of mice

*Indicated significant discrepancy in the copulation plug rate between the AQP1 (−/−) and AQP1 (+/+) groups, P < 0.01

Data were compared by Chi-square test, values are reported as n (%)

The number of mice in each of the four groups of atrophied embryos rate was 12

**Fig. 5:**
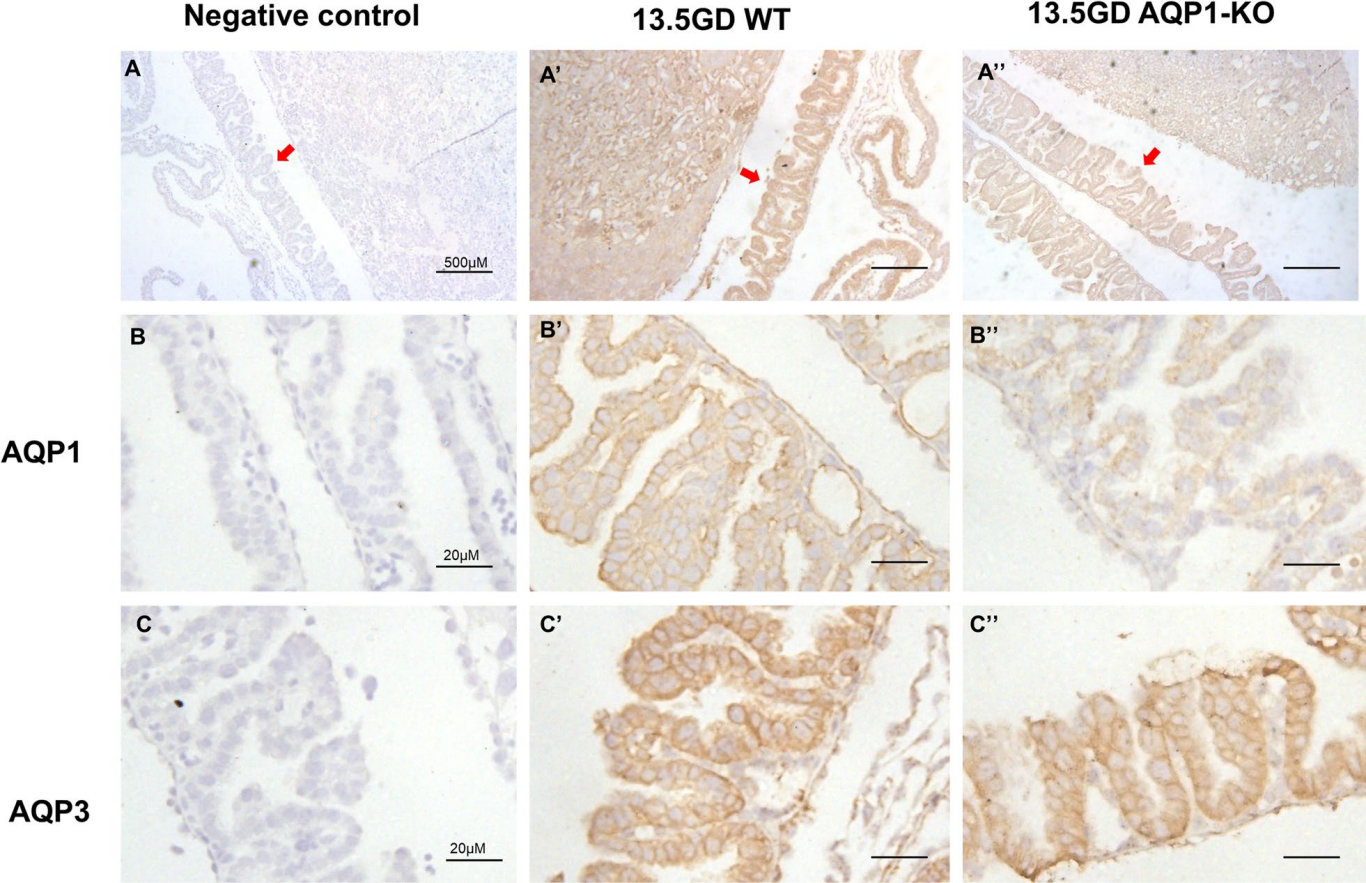
Localization of AQPs in 13.5 GD mouse fetal membrane. **A** Mouse fetal membrane near the placenta. **A’, A’’** gave an overview of AQP1 and AQP3 immunohistochemical staining in mouse fetal membrane of WT and AQP1-KO. Scale bars, 500 μm. **B, C** Fetal membrane epithelial cells. **B’, C’** AQP1 and AQP3 were expressed in the cell membrane of fetal membrane epithelium of WT mice. **B’’** Weak immunohistochemical staining of AQP1 were observed in fetal membrane of AQP1-KO mice. **C’’** AQP3 expression in fetal membrane did not change significantly at AQP1-KO mice. Scale bars, 20 μm. Negative controls used PBS instead of primary antibody

**Fig. 6:**
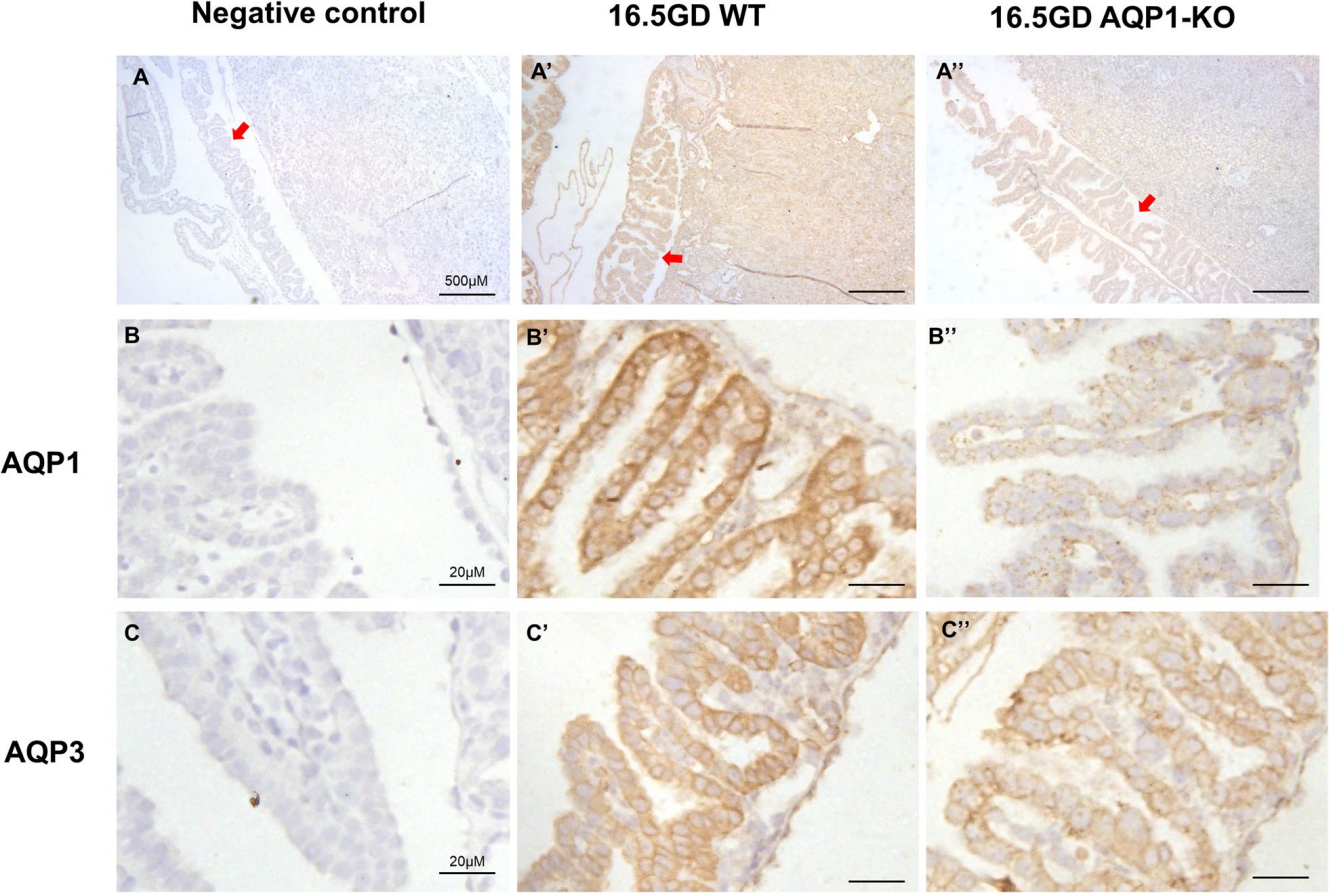
Localization of AQPs in 16.5 GD mouse fetal membrane. (A) Mouse fetal membrane near the placenta. **A’, A’’** gave an overview of AQP1 and AQP3 immunohistochemical staining in mouse fetal membrane of WT and AQP1-KO.Scale bars, 500 μm. **B, C** Fetal membrane epithelial cells. **B’, C’** AQP1 and AQP3 were expressed in the cell membrane of fetal membrane epithelium of WT mice. **B’’** Weak immunohistochemical staining of AQP1 were detected in fetal membrane of AQP1-KO mice. **C’’** AQP3 expression in fetal membrane did not change significantly at AQP1-KO mice. Scale bars, 20 μm. Negative controls used PBS instead of primary antibody

**Fig. 7:**
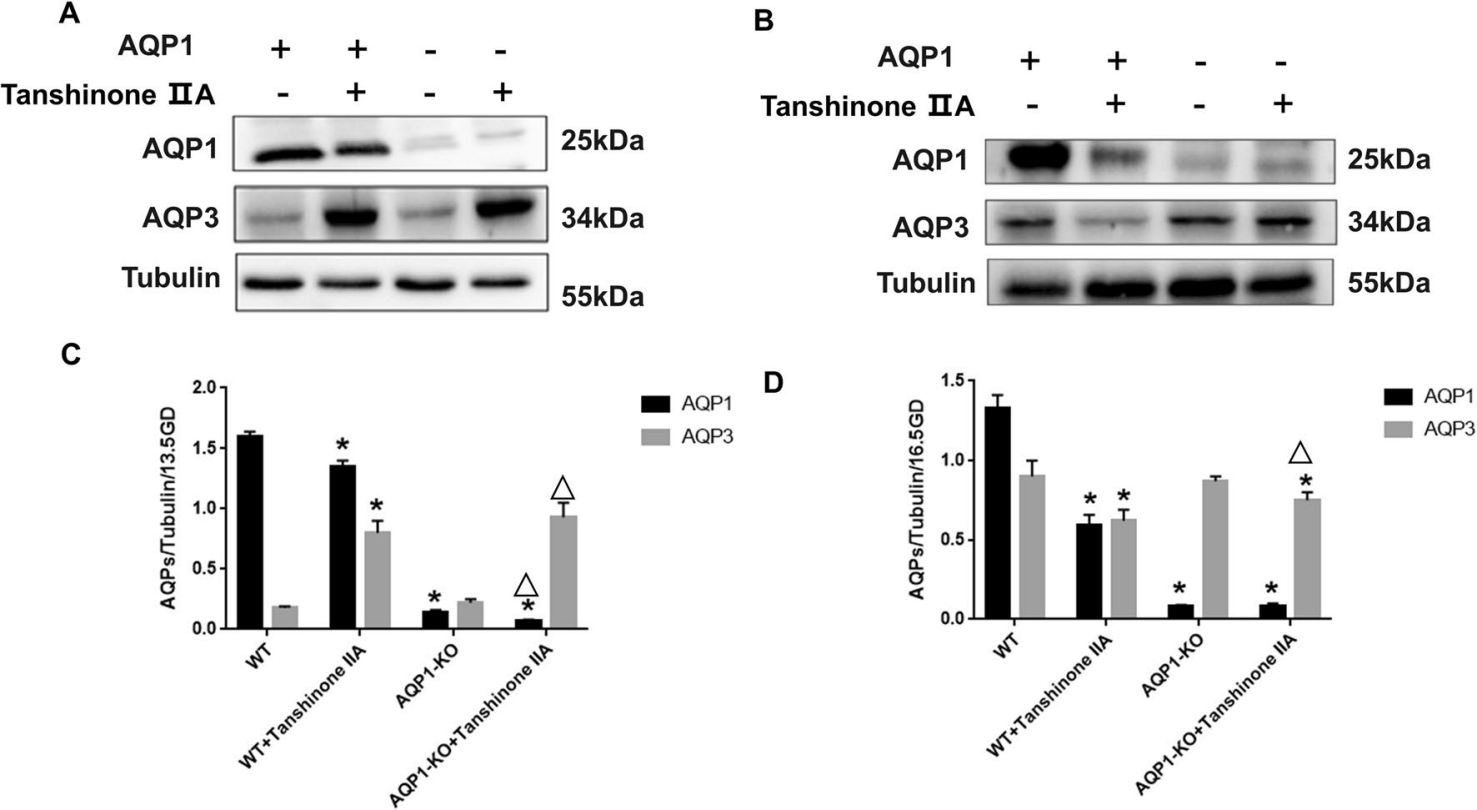
Tanshinone IIA regulated the expression of AQP1 and AQP3 proteins in the fetal membranes of pregnancy mice at 13.5GD and 16.5GD. **A** Western blotting showed that expression of AQP1, AQP3 protein of mice fetal membranes in WT group, WT + Tanshinone IIA group, AQP1-KO group, and AQP1-KO + Tanshinone IIA group in 13.5GD. **B **Western blotting showed that expression of AQP1, AQP3 protein of mice fetal membranes in each group in 16.5GD. **A’, B’** Quantification of the expressions of AQP1, AQP3 relative to Tubulin. * and △ represent significant difference compared with WT group and AQP1-KO group, respectively, *P* < 0.05

AQPs staining were detected in fetal membrane epithelial cells of AQP1-WT and AQP1-KO mice by Immunohistochemistry at 13.5GD and 16.5GD, respectively. AQP1 and AQP3 were localized in the cell membrane of fetal membrane epithelium. Weak immunohistochemical staining of AQP1 was observed in the fetal membrane epithelial cells of AQP1-KO mice (Figs. 5, 6). Additionally, AQP3 expression in mouse fetal membrane did not change significantly after AQP1 knockdown (Figs. 5, 6, 7).

Therefore, AQP1-KO mice showed an increased amniotic fluid volume and a decreased copulation plug rate, but the AQP1-KO did not affect the successful pregnancy rates, fetal weight and placental weight in pregnant mice.

### Tanshinone IIA regulated AQP1 and AQP3 protein expression in the fetal membrane in vivo

Tanshinone IIA showed different regulatory effects on AQP3 expression in fetal membranes between 13.5GD and 16.5GD whether in wild-type mice or AQP1 knockout mice. At 13.5GD, AQP3 expression was higher in Tanshinone IIA group than in control group, while it was lower in Tanshinone IIA group vs control group at 16.5GD whether in wild-type mice or AQP1-KO mice (Fig. 7). Compared to the control group, Tanshinone IIA consistently down-regulated the expression of AQP1 in amniotic membrane of WT mice and AQP1-KO mice at 13.5GD (Fig. 7). Therefore, Tanshinone IIA decreased AQP1 and AQP3 protein expression in the fetal membrane of pregnant mice at 16.5GD.

### Tanshinone IIA affected the AFV, fetal weight and placental weight in mice

**Fig. 8:**
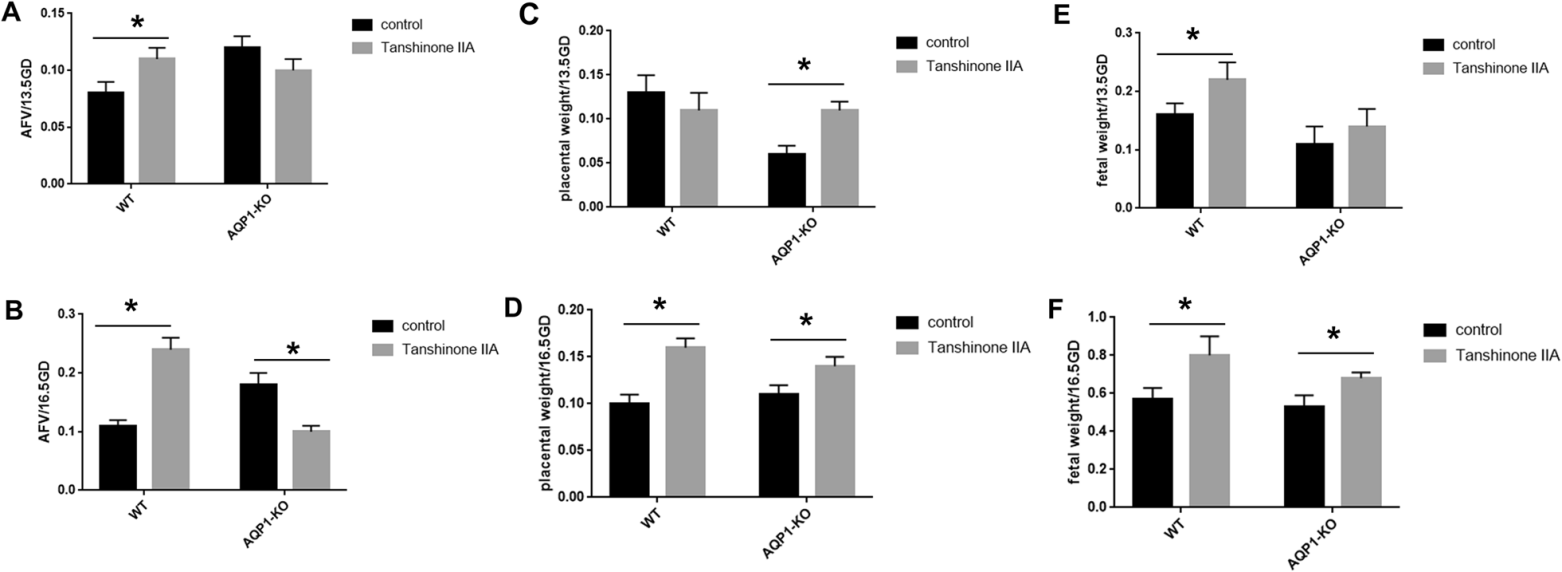
Tanshinone IIA affected the AFV, fetal weight and placental weight in WT mice and AQP1-KO mice. **A, C, E** AFV, fetal weight, and placental weight in WT mice and AQP1-KO mice at 13.5GD. **B, D, E** AFV, fetal weight, and placental weight in WT mice and AQP1-KO mice at 16.5GD. * represents significant difference compared with control group and Tanshinone IIA group, *P* < 0.05. *AFV* amniotic fluid volume

To investigate the effect of Tanshinone IIA on AQP1-KO mice and wild-type mice, the pregnant mice were intraperitoneally administrated with Tanshinone IIA (10 mg/kg) from 9.5GD to 13.5GD or 16.5GD. Compared with the control group, AFV, placental weight and fetal weight of wild-type mice in Tanshinone IIA group at 13.5GD and 16.5GD were significantly improved. For AQP1-KO mice, the fetal weight and placental weight in Tanshinone IIA group were significantly higher than those in control group, and AFV was significantly lower than that in control group at 16.5GD (Fig. 8). Therefore, Tanshinone IIA increased placental weight and fetal weight both in wild-type mice and AQP1-KO mice, but Tanshinone IIA increased AFV in wild-type mice and decreased AFV in AQP1-KO mice.

### Tanshinone IIA regulated AQP1 and AQP3 protein expression in hAECs in vitro

**Fig. 9:**
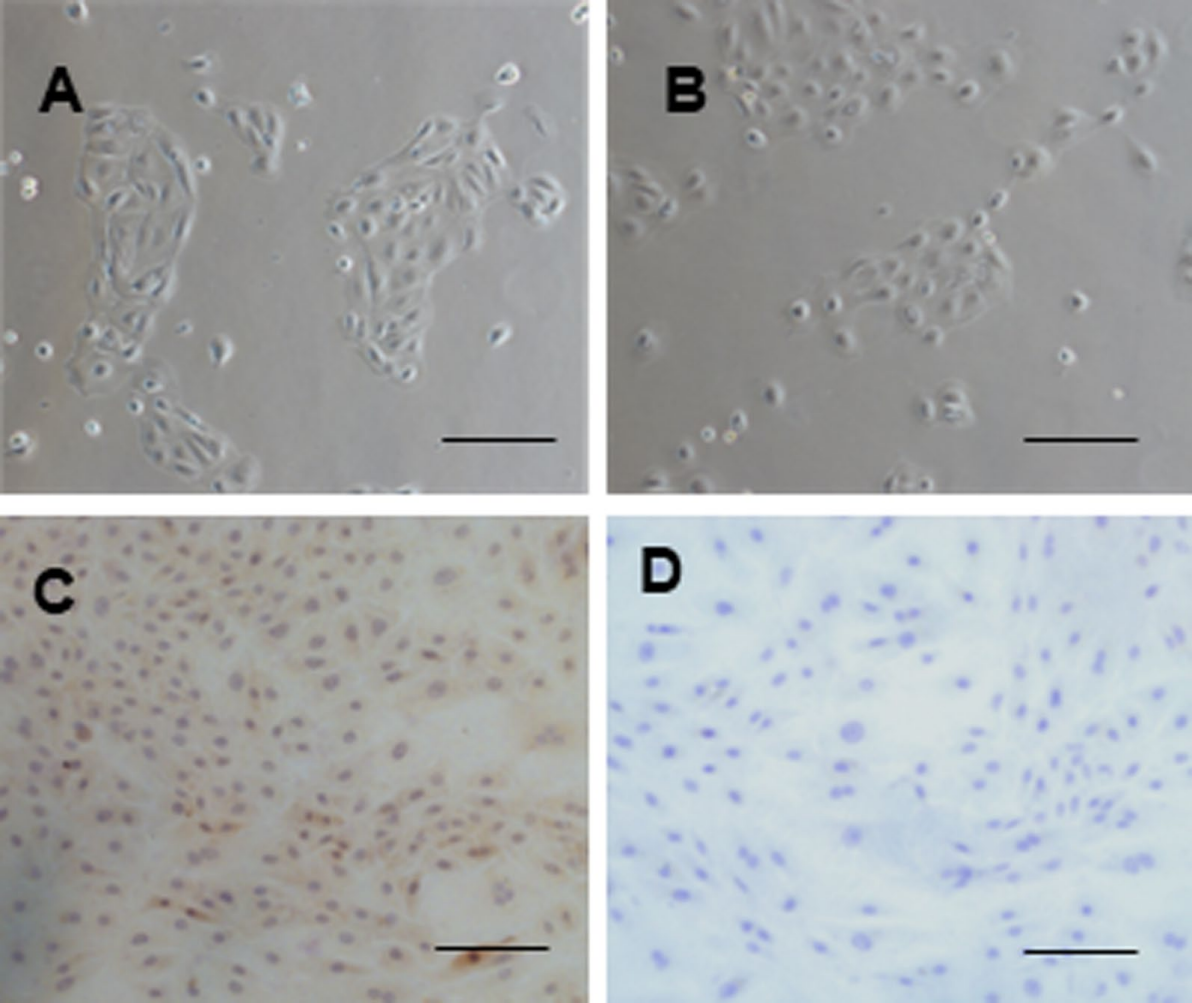
Identification of amniotic epithelial cells. Primary cultured human amniotic epithelial cells were shown by light microscopy. **A** Normal AFV group. **B** Isolated oligohydramnios. **C** Immunocytochemistry staining of CK-18 in hAECs. **D** Negative control used PBS instead of primary antibody. Bar, 20 μm. *hAECs* human amniotic epithelial cells

**Fig. 10:**
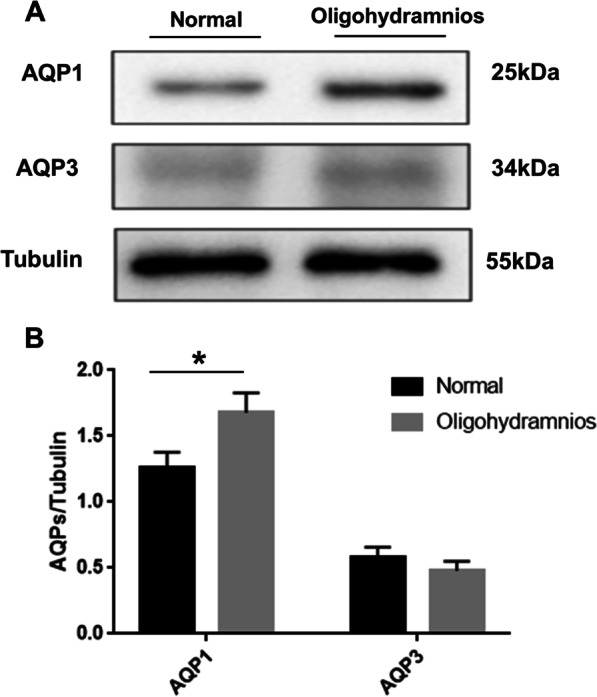
The expression of AQP1 on hAECs of oligohydramnios group was significantly higher than that in normal AFV group. **A** Western blotting of AQP1, AQP3 protein expression in hAECs. **B** Quantification of the expressions of AQP1, AQP3 relative to Tubulin. * represents significant difference compared with normal AFV group and isolated oligohydramnios group, *P* < 0.05. *hAECs* human amniotic epithelial cells

**Fig. 11:**
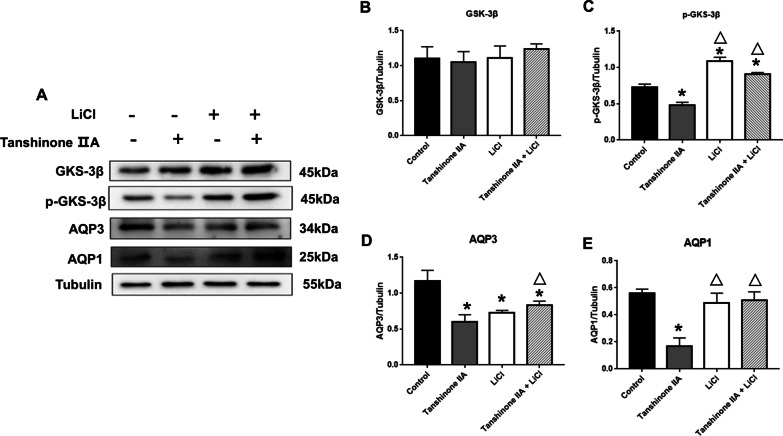
Tanshinone IIA reduced AQP1 and AQP3 expressions in normal hAECs via GSK-3β signaling pathway. **A** Western blotting of GSK-3β, p-GSK-3β, AQP1 and AQP3 proteins expression in hAECs with normal AFV. **B–E** Quantification of the expressions of GSK-3β, p-GSK-3β, AQP1, AQP3 relative to Tubulin. * and △ represent significant difference compared with control group and Tanshinone IIA group, respectively, *P* < 0.05. *hAECs* human amniotic epithelial cells, *AFV* amniotic fluid volume

**Fig. 12:**
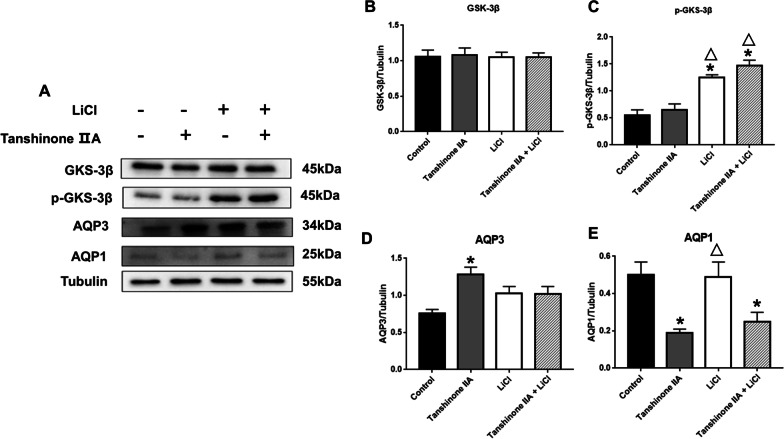
Tanshinone IIA regulated AQP1 and AQP3 expressions in hAECs of isolated oligohydramnios. **A** Western blotting of GSK-3β, p-GSK-3β, AQP1 and AQP3 proteins expression in hAECs with isolated oligohydramnios. **B–E** Quantification of the expressions of GSK-3β, p-GSK-3β, AQP1, AQP3 relative to Tubulin. * and △ represent significant difference compared with control group and Tanshinone IIA group, respectively, *P* < 0.05. *hAECs* human amniotic epithelial cells

To explore the relationship between AQP1 and AQP3 expression regulated by Tanshinone IIA and AFV, amniotic membrane was extracted from pregnant women for primary culture (Fig. 9). Compared with normal AFV group, AQP1 protein expression in hAECs was significantly increased in the isolated oligohydramnios group, while AQP3 protein expression was not significantly different (Fig. 10). The expression of AQP1 and AQP3 protein in hAECs from pregnant women with normal AFV were significantly reduced by Tanshinone IIA (Fig. 11). Interestingly, in hAECs from pregnant women with isolated oligohydramnios, Tanshinone IIA decreased AQP1 protein expression and increased expression of AQP3 (Fig. 12). Therefore, Tanshinone IIA decreased the expression of AQP1 and AQP3 in hAECs from pregnant women with normal AFV, and Tanshinone IIA decreased AQP1 protein expression and increased the AQP3 expression in hAECs from pregnant women with isolated oligohydramnios.

### Tanshinone IIA down-regulated AQP1 and AQP3 expressions in normal hAECs via GSK-3β signaling pathway

To explore the possible mechanism of Tanshinone IIA regulating AQPs expression, human amniotic cells were treated with GSK-3β inhibitor (LiCl). There was no significant difference in total GSK-3β protein expression in the hAECs of normal AFV pregnant women between the groups. Compared with the control group, Tanshinone IIA decreased expression of p-GSK-3β, AQP1 and AQP3, and LiCl significantly eliminate the reduction of p-GSK-3β, AQP1 and AQP3 protein expression mediated by Tanshinone IIA (Fig. 11).

In the hAECs of isolated oligohydramnios, the expression of total GSK-3β was not significantly affected by Tanshinone IIA and LiCl. The p-GSK-3β protein expression of isolated oligohydramnios cells was stimulated by LiCl, but was not affected by Tanshinone IIA. The expression of AQP3 protein increased and AQP1 protein decreased after treatment with Tanshinone IIA, while LiCl did not have a significant effect on their expression (Fig. 12).

Therefore, Tanshinone IIA reduced AQP1 and AQP3 protein expression in hAECs from normal AFV pregnant women by inhibiting the activity of GSK-3β, but the effect of Tanshinone IIA on the expression of AQP3 and AQP1 in hAECs with isolated oligohydramnios were not associated with GSK-3β signaling pathway.

## Discussion

The intramembrane absorption of amniotic fluid plays a vital role in maintaining normal amniotic fluid volume, and AQPs are channel proteins expressed in mammalian amniotic membranes. In this study, we found that the expression of AQP1 protein in amniotic membrane was significantly increased in pregnant women with isolated oligohydramnios and there was no difference in AQP3 protein expression in amniotic membrane between normal AFV and isolated oligohydramnios. Therefore, we hypothesized that the abnormal expression of AQP1 in amniotic membrane may lead to the abnormal AFV, and regulation of AQP1 expression in amniotic membrane may be a potential treatment for abnormal AFV.

To further explore the relationship between AQPs and abnormal AFV, we compared WT mice and AQP1-KO mice. It had been found that AQP1 knockout resulted in a significant increase in AFV and a decrease in the copulation plug rate in mice. Likewise, Mann, et al. used AQP1 knockout mice to provide a novel animal model of polyhydramnios in 2005 (Mann et al. 2005). Zheng et al. (2014) and Luo et al. (2020) also found that AQP1 knockout mice showed significantly increased amniotic fluid volume and decreased osmolality of amniotic fluid compared to wild-type mice. Therefore, the downregulation of AQP1 protein expression can lead to increased amniotic fluid volume.

A clinical trial has shown that salvia miltiorrhiza is an effective medicine for oligohydramnios (Chu and Shen 2008). Tanshinone IIA is the active ingredient extracted from the Salvia miltiorrhiza. Our previous studies demonstrated that Tanshinone IIA could increase amniotic fluid volume of pregnancy mice (Shao et al. 2022). In current study, we observed that Tanshinone IIA significantly increased the amniotic fluid volume in wild-type mice, not in AQP1 gene knockout mice. In addition, Tanshinone IIA reduced AQP1 protein expression in the fetal membranes of wild-type mice and in human amniotic epithelial cells from the normal AFV pregnant women. Thus, we suggest that Tanshinone IIA may increase AFV in wild-type mice by downregulating AQP1 protein expression in the fetal membranes.

However, we found Tanshinone IIA did not further augment the increased amniotic fluid volume caused by AQP1 knockout. Instead, it reduced amniotic fluid volume in AQP1 knockout mice. It is difficult to explain this. It may be due to compensatory alterations of other AQPs in the fetal membranes induced by AQP1 knock-down, such as AQP3, AQP8 and AQP9 expression (Beall et al. 2007b). AQP3 knockout mice exhibited the reduced AFV (Seo et al. 2018). Our study found that Tanshinone IIA reduced the expression of AQP3 in fetal membranes of AQP1-KO pregnancy mice during third trimester. Besides, in human amniotic epithelial cells of the isolated oligohydramnios, Tanshinone IIA can increase the AQP3 protein expression. Also, salvia miltiorrhiza extract is effective in treating oligohydramnios clinically, and it was able to increase AQP3 expression in amniotic epithelial cells (Zhang et al. 2017).

Thus, a larger AFV in AQP1-KO mice was significantly attenuated by Tanshinone IIA, probably because Tanshinone IIA regulates AQP3 protein expression in fetal membranes. Tanshinone IIA changes amniotic fluid volume by its regulation of different AQPs, thereby maintaining AFV in a normal stable state. Tanshinone IIA may improve abnormal amniotic fluid volume and treat oligohydramnios, but it did not increase amniotic fluid volume unrestrictively by regulating the expression of different AQPs in the fetal membrane. Therefore, Tanshinone IIA is safe for the treatment of oligohydramnios and does not lead to polyhydramnios.

Except for water, AQP3 can transport glycerol and other nutrients (Shao et al. 2021). Seo et al. found that fetal weight was significantly lower in AQP3 knockout mice compared to wild-type mice (Seo et al. 2018). Our data showed that AQP3 protein expression in fetal membranes of in AQP1-KO mice did not differ compared to WT mice, as well as no significant difference in fetal weight. This suggested that AQP1 did not affect fetal weight, but alteration of AQP3 expression may be related to fetal weight. Futhermore, we found that Tanshinone IIA increased fetal and placental weight in both WT mice and AQP1-KO mice, which is related to the increased expression of AQP3 to some extent. Thus, AQP3 expression regulated by Tanshinone IIA is closely associated with fetal weight, but the possible molecular mechanism needs to be further studied.

It was reported that Tanshinone IIA taked the remarkable organoprotective effects at the molecular level via targeting GSK-3β (Jiang et al. 2016; Lin et al. 2019; Sun et al. 2011). As a serine/threonine protein kinase, GSK-3β plays an important role in various biological processes including cell proliferation, DNA repair and metabolic pathways (Lin et al. 2020; Takahashi-Yanaga and Sasaguri 2009). LiCl promotes phosphorylation of Ser9 on GSK-3β protein thereby rendering it inactive, but had no effect on total GSK-3β protein expression (King et al. 2014; Ebeid et al. 2021). In the current study, total GSK-3β protein expression in the human amniotic epithelial cells did not differ among groups, whether from normal AFV pregnant women or isolated oligohydramnios pregnant women. But Tanshinone IIA decreased p-GSK-3β, as well as AQP1 and AQP3 protein expression in human amniotic epithelial cells of normal AFV pregnant women, which was significantly improved by LiCl. These data indicated that Tanshinone IIA may regulate AQP1 and AQP3 protein expression in normal AFV cells through activating GSK-3β signaling pathway. Therefore, we suggested that Tanshinone IIA reduced AQP1 protein expression through the GSK-3 signaling pathway, ultimately increasing the AFV in normal pregnancies.

However, in isolated oligohydramnios cells, the p-GSK-3β protein expression was not affected by Tanshinone IIA, and the AQPs protein expression was not affected by LiCl, suggesting that the regulation of AQPs expression by Tanshinone IIA in the amniotic epithelial cells of isolated oligohydramnios is independent of GSK-3β. We observed that Tanshinone IIA reduced AQP1 and AQP3 protein expression in hAECs from normal AFV pregnant women, while AQP1 and AQP3 show reciprocal relationship after Tanshinone IIA treatment in the oligohydramnios. Furthermore, compared with pregnant women the normal AFV, AQP1 expression was increased in the amniotic membrane of pregnant women with oligohydramnios, but no significant difference was found in AQP3 expression. So we suggested that the effect of Tanshinone IIA on AQP1 and AQP3 varied in physiological and pathological conditions. Likewise, the molecular mechanism of regulating AQPs protein expression under the pathological conditions of oligohydramnios is intricate and esoteric, for example, changes in upstream and downstream proteins of pathway. Further study was needed to conduct to investigate the molecular mechanisms of its regulation of AQPs protein expression in the pathological condition of oligohydramnios.

## Conclusions

To sum up, AQP1 knockout led to increased amniotic fluid volume. Tanshinone IIA may increase AFV in wild-type mice by downregulating AQP1 protein expression in the fetal membranes, which was related to GSK-3β signaling pathway. But Tanshinone IIA reduced the amount of amniotic fluid in AQP1 knockout mice, which may be due to compensatory alterations of other AQPs in the fetal membranes, like reduced AQP3 protein expression induced by Tanshinone IIA. Moreover, the regulation of AQPs expression by Tanshinone IIA in the amniotic epithelial cells of isolated oligohydramnios is independent of GSK-3β. In addition, Tanshinone IIA increased fetal weight, which may be related to alteration of AQP3 expression in fetal membrane.

## Data Availability

The datasets used and/or analysed during the current study are available from the corresponding author on reasonable request.

## Abbreviations

AFI: Amniotic fluid index
AFV: Amniotic fluid volume
AGE: Agarose gel electrophoresis
AQPs: Aquaporins
AQP1: Aquaporin1
AQP3: Aquaporin3
AQP1-KO: Aquaporin1 knockout
BCA: Bicinchoninic acid
DMSO: Dimethylsulfoxide
DMEM: Dulbecco’ modified eagle medium
EDTA: Ethylenediamine tetraacetic acid disodium
GD: Gestational days
GSK-3β: Glycogen synthetic kinase-3β
hAECs: Human amniotic epithelium cells
p-GSK-3β: Phospho-glycogen synthetic kinase-3β
PVDF: Polyvinylidene difluoride
SDS-PAGE: Sdium dodecyl sulfate polyacrylamide gel electrophoresis
SDP: Single deepest fluid pocket
WT mice: Wild-type mice

## References

[CR1] Ansari MA, Khan FB, Safdari HA, Almatroudi A, Alzohairy MA, Safdari M (2021). Prospective therapeutic potential of Tanshinone IIA: an updated overview. Pharmacol Res.

[CR2] Aralla M, Mobasheri A, Groppetti D, Cremonesi F, Arrighi S (2012). Expression of aquaporin water channels in canine fetal adnexa in respect to the regulation of amniotic fluid production and absorption. Placenta.

[CR3] Beall MH, van den Wijngaard JP, van Gemert MJ, Ross MG (2007). Amniotic fluid water dynamics. Placenta.

[CR4] Beall MH, Wang S, Yang B, Chaudhri N, Amidi F, Ross MG (2007). Placental and membrane aquaporin water channels: correlation with amniotic fluid volume and composition. Placenta.

[CR5] Bednar AD, Beardall MK, Brace RA, Cheung CY (2015). Differential expression and regional distribution of aquaporins in amnion of normal and gestational diabetic pregnancies. Physiol Rep.

[CR6] Carbrey JM, Agre P (2009). Discovery of the aquaporins and development of the field. Handb Exp Pharmacol.

[CR7] Choi HJ, Cha SJ, Lee JW, Kim HJ, Kim K (2020). Recent advances on the role of gsk3beta in the pathogenesis of amyotrophic lateral sclerosis. Brain Sci.

[CR8] Chu HN, Shen MJ (2008). Treating oligohydramnios with extract of Salvia miltiorrhiza: a randomized control trial. Ther Clin Risk Manag.

[CR9] Croom CS, Banias BB, Ramos-Santos E, Devoe LD, Bezhadian A, Hiett AK (1992). Do semiquantitative amniotic fluid indexes reflect actual volume?. Am J Obstet Gynecol.

[CR10] Damiano A, Zotta E, Goldstein J, Reisin I, Ibarra C (2001). Water channel proteins AQP3 and AQP9 are present in syncytiotrophoblast of human term placenta. Placenta.

[CR11] Ebeid MA, Habib MZ, Mohamed AM, Faramawy YE, Saad SST, El-Kharashi OA (2021). Cognitive effects of the GSK-3 inhibitor “lithium” in LPS/chronic mild stress rat model of depression: hippocampal and cortical neuroinflammation and tauopathy. Neurotoxicology.

[CR12] Hua Y, Ding S, Cheng H, Luo H, Zhu X (2017). Tanshinone IIA increases aquaporins expression in human amniotic epithelial WISH cells by stimulating GSK-3beta phosphorylation. Clin Chim Acta.

[CR13] Huang Y, Long X, Tang J, Li X, Zhang X, Luo C (2020). The attenuation of traumatic brain injury via inhibition of oxidative stress and apoptosis by tanshinone IIA. Oxid Med Cell Longev.

[CR14] Jiang C, Zhu W, Yan X, Shao Q, Xu B, Zhang M (2016). Rescue therapy with Tanshinone IIA hinders transition of acute kidney injury to chronic kidney disease via targeting GSK3beta. Sci Rep.

[CR15] King MK, Pardo M, Cheng Y, Downey K, Jope RS, Beurel E (2014). Glycogen synthase kinase-3 inhibitors: rescuers of cognitive impairments. Pharmacol Ther.

[CR16] Li J, Xu M, Fan Q, Xie X, Zhang Y, Mu D (2011). Tanshinone IIA ameliorates seawater exposure-induced lung injury by inhibiting aquaporins (AQP) 1 and AQP5 expression in lung. Respir Physiol Neurobiol.

[CR17] Lin L, Jadoon SS, Liu SZ, Zhang RY, Li F, Zhang MY (2019). Tanshinone IIA ameliorates spatial learning and memory deficits by inhibiting the activity of ERK and GSK-3beta. J Geriatr Psychiatry Neurol.

[CR18] Lin J, Song T, Li C, Mao W (2020). GSK-3beta in DNA repair, apoptosis, and resistance of chemotherapy, radiotherapy of cancer. Biochim Biophys Acta Mol Cell Res.

[CR19] Luo H, Xie A, Hua Y, Wang J, Liu Y, Zhu X (2018). Aquaporin 1 gene deletion affects the amniotic fluid volume and composition as well as the expression of other aquaporin water channels in placenta and fetal membranes. Clin Chim Acta.

[CR20] Luo H, Liu Y, Song Y, Hua Y, Zhu X (2020). Aquaporin 1 affects pregnancy outcome and regulates aquaporin 8 and 9 expressions in the placenta. Cell Tissue Res.

[CR21] Mann SE, Ricke EA, Yang BA, Verkman AS, Taylor RN (2002). Expression and localization of aquaporin 1 and 3 in human fetal membranes. Am J Obstet Gynecol.

[CR22] Mann SE, Ricke EA, Torres EA, Taylor RN (2005). A novel model of polyhydramnios: amniotic fluid volume is increased in aquaporin 1 knockout mice. Am J Obstet Gynecol.

[CR23] Mann SE, Dvorak N, Gilbert H, Taylor RN (2006). Steady-state levels of aquaporin 1 mRNA expression are increased in idiopathic polyhydramnios. Am J Obstet Gynecol.

[CR24] Ozgen G, Dincgez Cakmak B, Ozgen L, Uguz S, Sager H (2021). The role of oligohydramnios and fetal growth restriction in adverse pregnancy outcomes in preeclamptic patients. Ginekol Pol.

[CR25] Pérez-Pérez A, Vilariño-García T, Dietrich V, Guadix P, Dueñas JL, Varone CL (2020). Aquaporins and placenta. Vitam Horm.

[CR26] Quan M, Lv Y, Dai Y, Qi B, Fu L, Chen X (2019). Tanshinone IIA protects against lipopolysaccharide-induced lung injury through targeting Sirt1. J Pharm Pharmacol.

[CR27] Seo MJ, Lim JH, Kim DH, Bae HR (2018). Loss of aquaporin-3 in placenta and fetal membranes induces growth restriction in mice. Dev Reprod.

[CR28] Shao H, Gao S, Ying X, Zhu X, Hua Y (2021). Expression and regulation of aquaporins in pregnancy complications and reproductive dysfunctions. DNA Cell Biol.

[CR29] Shao H, Pan S, Lan Y, Chen X, Dai D, Peng L (2022). Tanshinone IIA increased amniotic fluid volume through down-regulating placental AQPs expression via inhibiting the activity of GSK-3beta. Cell Tissue Res.

[CR30] Sun D, Shen M, Li J, Li W, Zhang Y, Zhao L (2011). Cardioprotective effects of tanshinone IIA pretreatment via kinin B2 receptor-Akt-GSK-3beta dependent pathway in experimental diabetic cardiomyopathy. Cardiovasc Diabetol.

[CR31] Takahashi-Yanaga F, Sasaguri T (2009). Drug development targeting the glycogen synthase kinase-3beta (GSK-3beta)-mediated signal transduction pathway: inhibitors of the Wnt/beta-catenin signaling pathway as novel anticancer drugs. J Pharmacol Sci.

[CR32] Yan P, Xu D, Ji Y, Yin F, Cui J, Su R (2019). LiCl pretreatment ameliorates adolescent methamphetamine exposure-induced long-term alterations in behavior and hippocampal ultrastructure in adulthood in mice. Int J Neuropsychopharmacol.

[CR33] Zhang W, Zhang Y, Hu X, Cheng H, Hua Y, Zhu X (2017). Danshen extract regulates the expression of aquaporin 3 in human amniotic epithelial cells. Front Biosci (landmark Ed).

[CR34] Zheng Z, Liu H, Beall M, Ma T, Hao R, Ross MG (2014). Role of aquaporin 1 in fetal fluid homeostasis. J Matern Fetal Neonatal Med.

[CR35] Zhou L, Zuo Z, Chow MS (2005). Danshen: an overview of its chemistry, pharmacology, pharmacokinetics, and clinical use. J Clin Pharmacol.

[CR36] Zhu C, Jiang Z, Bazer FW, Johnson GA, Burghardt RC, Wu G (2015). Aquaporins in the female reproductive system of mammals. Front Biosci (landmark Ed).

